# Supplementary material for: Correcting modification-mediated errors in nanopore sequencing by nucleotide demodification and reference-based correction
Source: Commun Biol. 2023 Nov 29;6:1215. doi: 10.1038/s42003-023-05605-4 (PMC10687267; doi:10.1038/s42003-023-05605-4)
Supplement: Supplementary file 1 — Supplementary Information [file 42003_2023_5605_MOESM1_ESM.pdf]

Supplementary Figures and Tables

Table of Contents

Supplementary Figure 1. Examples of ONT modification-mediated errors revealed by IGV. 3

Supplementary Figure 2. Comparison of ONT-only and WGA-demodified ONT genome quality at different sequencing depth: R19-2904, R20-0026, R20-0030, R20-0088, R20-0127, and R20-0131. 4

Supplementary Figure 3. Comparison of ONT-only and WGA-demodified ONT genome quality at different sequencing depth: R20-0140, R20-0145, R20-0148, R20-0150, R20-0158, and R20-0160. 5

Supplementary Figure 4. Comparison of base qualities between modification-mediated errors and modification-free loci. 6

Supplementary Figure 5. Illustration of double-stranded modification by IGV 6

Supplementary Figure 6. Comparison of the modification motifs for the ST1081 and ST81 strains separately on the hybrid ONT/Illumina and ONT-only genomes. 7

Supplementary Figure 7. Comparison of the mismatches and Q scores of Medaka and Modpolish in the 12 *Listeria* strains basecalled by Guppy 6.3.4 using the SUP model. 7

Supplementary Figure 8. BLAST search of the mza system members revealed a DNA cytosine methyltransferase followed by mzaB, mzaC, mzaD, and mzaE. 8

Supplementary Figure 9. Comparison of mismatches and Q scores of Medaka and Modpolish using the R10.4 simplex dataset (kit 14, Dorado v0.1.1, SUP). 8

Supplementary Figure 10. The numbers of mismatches and Q scores in the *E coli* genomes polished by Medaka (red), Homopolish (blue), and Modpolish (organge). 9

Supplementary Figure 11. Comparison of the cgMLST phylogeny of the five LQ *L monocytogene* strains among different methods. 9

Supplementary Figure 12. Similarity computation and allele count statistics. 10

Supplementary Table 1. Illumina sequencing statistics of the 12 samples. 11

Supplementary Table 2. ONT sequencing statistics of the 12 samples (R9.4, SUP). 11

Supplementary Table 3. Assembly statistics of ONT-only sequencing (R9.4, SUP). 11

Supplementary Table 4. Assembly statistics of hybrid ONT/Illumina sequencing. 12

Supplementary Table 5. Quality assessment of ONT-only genomes polished by Medaka and Homopolish (R9.4, SUP). 12

Supplementary Table 6. Numbers of 5mC and 6mA modifications identified by Megalodon. 12

Supplementary Table 7. ONT sequencing statistics of the 12 WGA-demodified samples. 13
Supplementary Table 8. Assembly statistics of WGA-demodified ONT sequencing 13
Supplementary Table 9. Quality assessment of WGA-demodified ONT genomes 13
Supplementary Table 10. Quality assessment of ONT genomes corrected by Modpolish. 14
Supplementary Table 11. The coverage, true positive (TP), true negative (TN), false positive (FP), false negative (FN), sensitivity, and specificity of Modpolish corrections on the 12 Listeria strains. 14
Supplementary Table 12. The true positive (TP), true negative (TN), false positive (FP), false negative (FN), sensitivity, and specificity of Modpolish on the Zymo R9.4 datasets. 15
Supplementary Table 14. Quality assessment of Zymo R9.4 dataset corrected by Modpolish 16
Supplementary Table 15. Quality assessment of Zymo R10.4 dataset corrected by Modpolish. 16
Supplementary Table 16. Comparison of contig numbers and N50 sizes of Illumina (ILMN), ONT WGS, and ONT WGA. 16

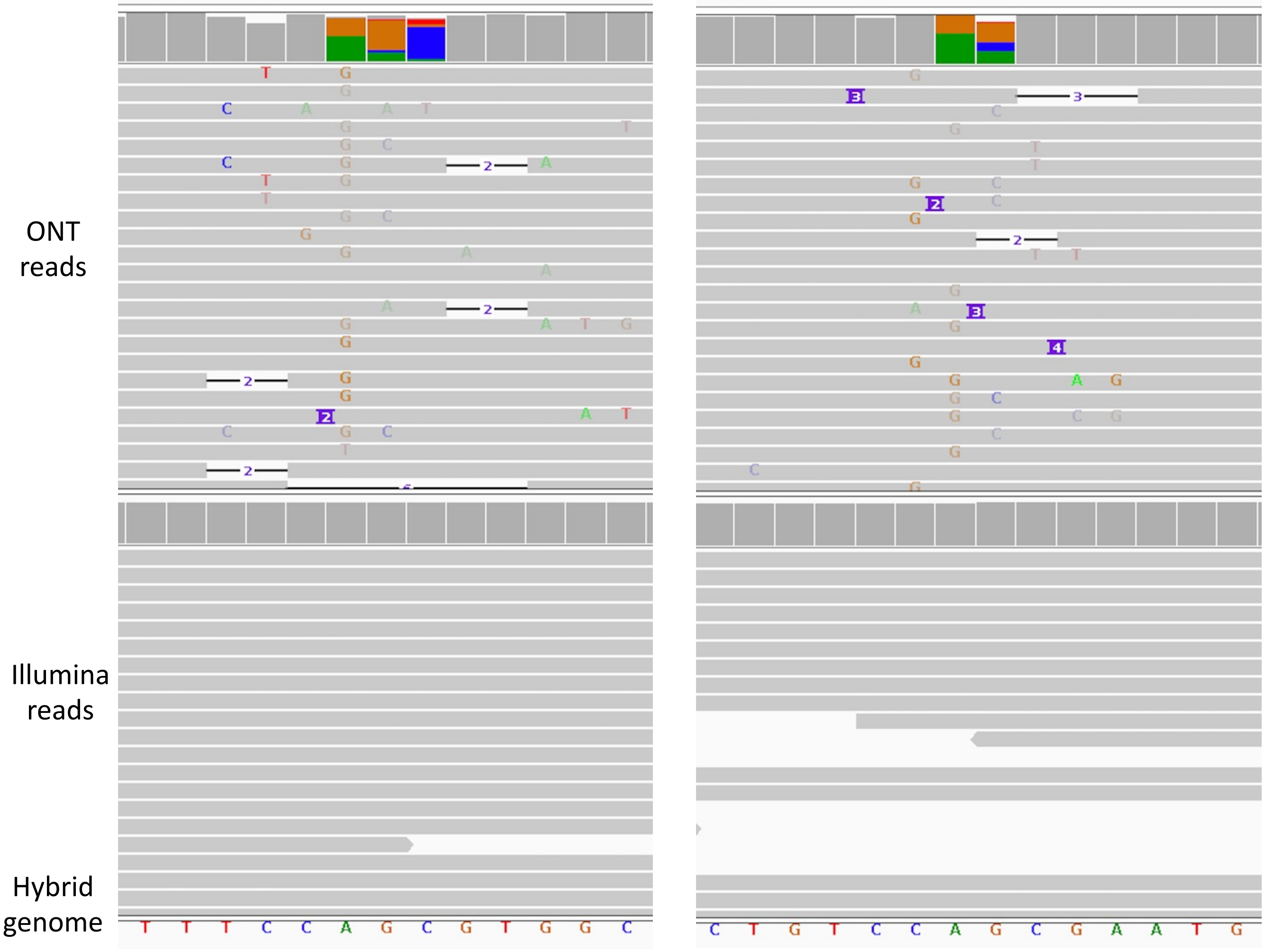

Supplementary Figure 1. Examples of ONT modification-mediated errors revealed by IGV. Top track: ONT reads; Middle track: Illumina reads; Bottom track: Hybrid assembled genome.

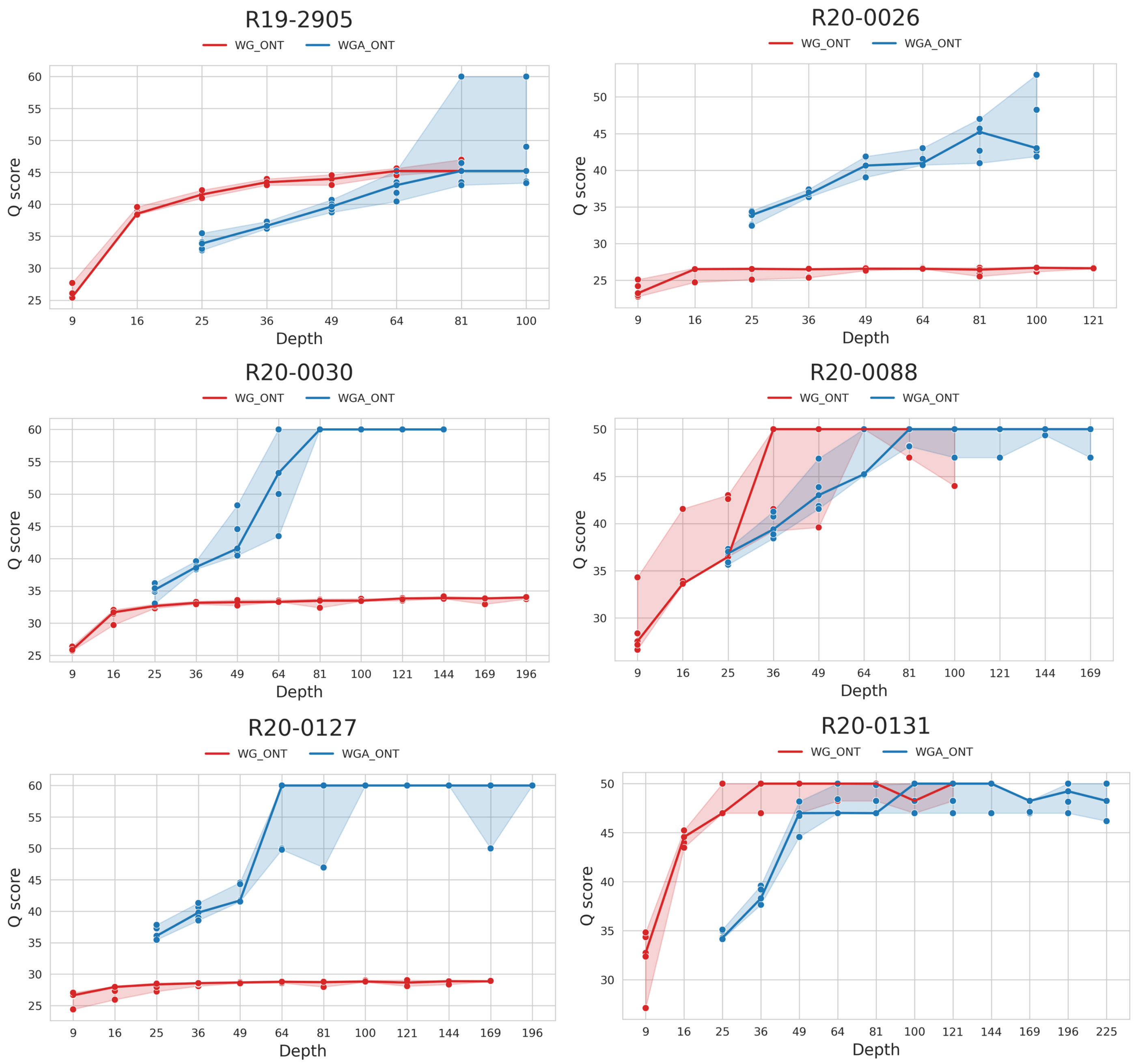

Supplementary Figure 2. Comparison of ONT-only and WGA-demodified ONT genome quality at different sequencing depth: R19-2904, R20-0026, R20-0030, R20-0088, R20-0127, and R20-0131. The shading spans the minimum and maximum values in the five replicated experiments (red: ONT, blue: WGA ONT).

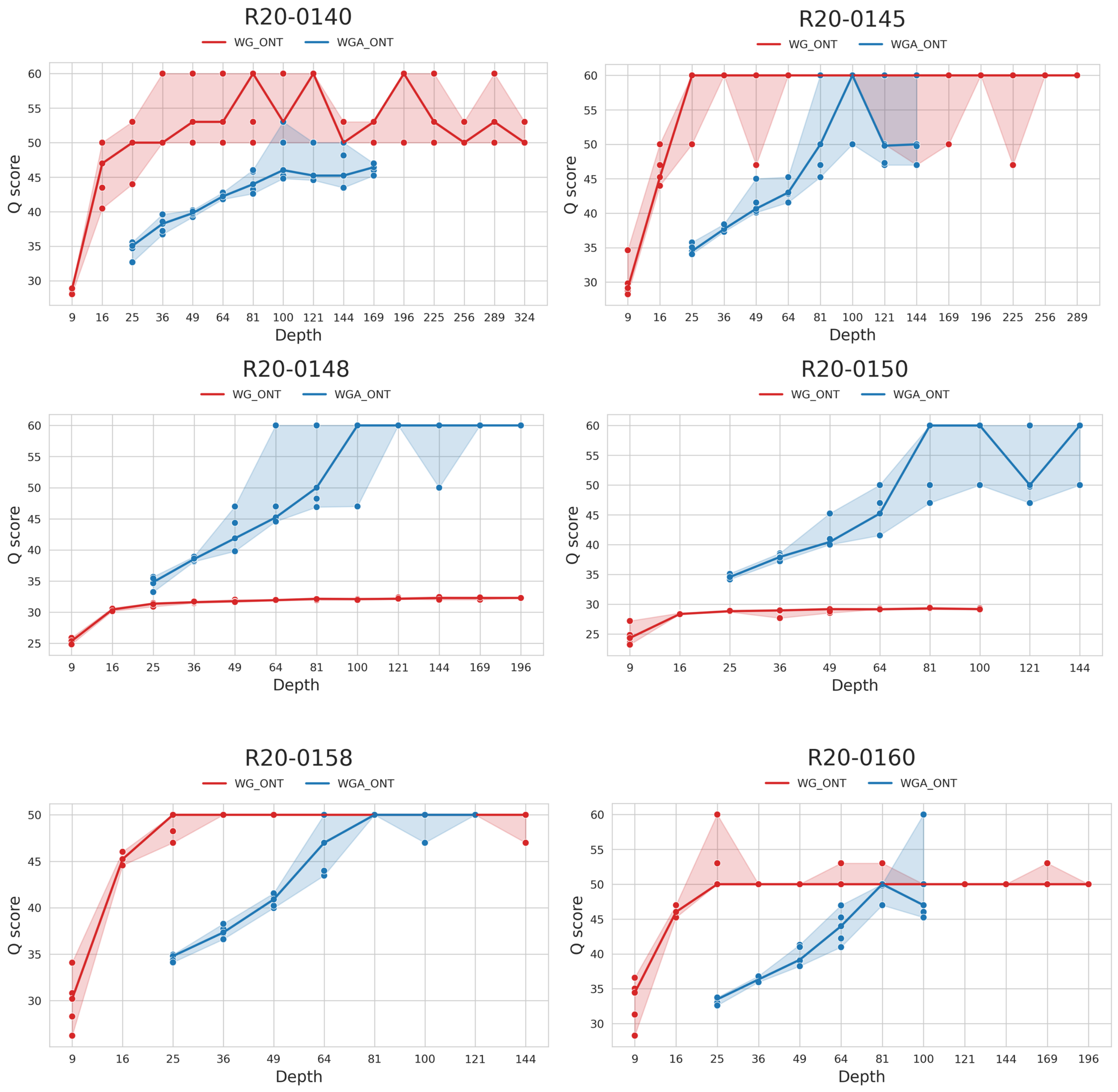

Supplementary Figure 3. Comparison of ONT-only and WGA-demodified ONT genome quality at different sequencing depth: R20-0140, R20-0145, R20-0148, R20-0150, R20-0158, and R20-0160. The shading spans the minimum and maximum values in the five replicated experiments (red: ONT, blue: WGA ONT).

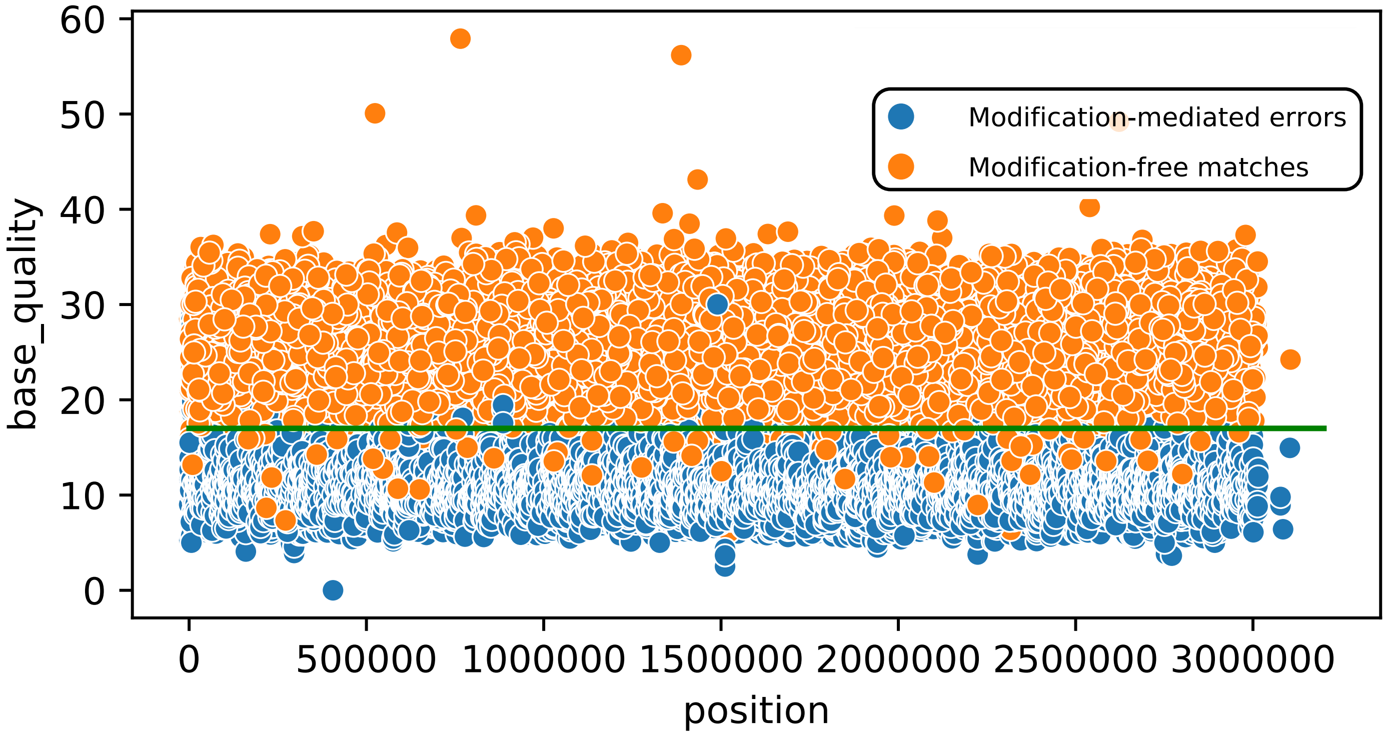

Supplementary Figure 4. Comparison of base qualities between modification-mediated errors and modification-free loci.

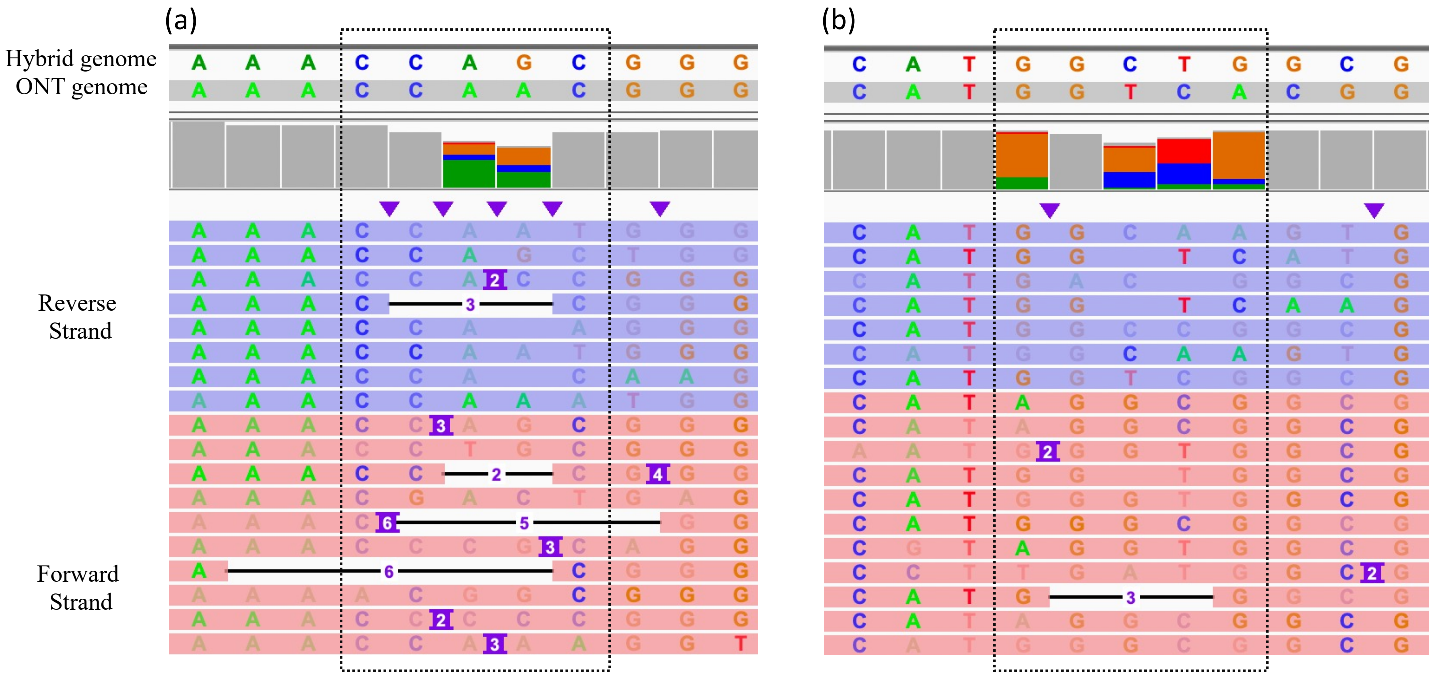

Supplementary Figure 5. Illustration of double-stranded modification by IGV. Basecalling errors were found on both the forward and reverse strands. (a) Sequencing errors for CCAGC motif; (b) sequencing errors for GGCTG motif.

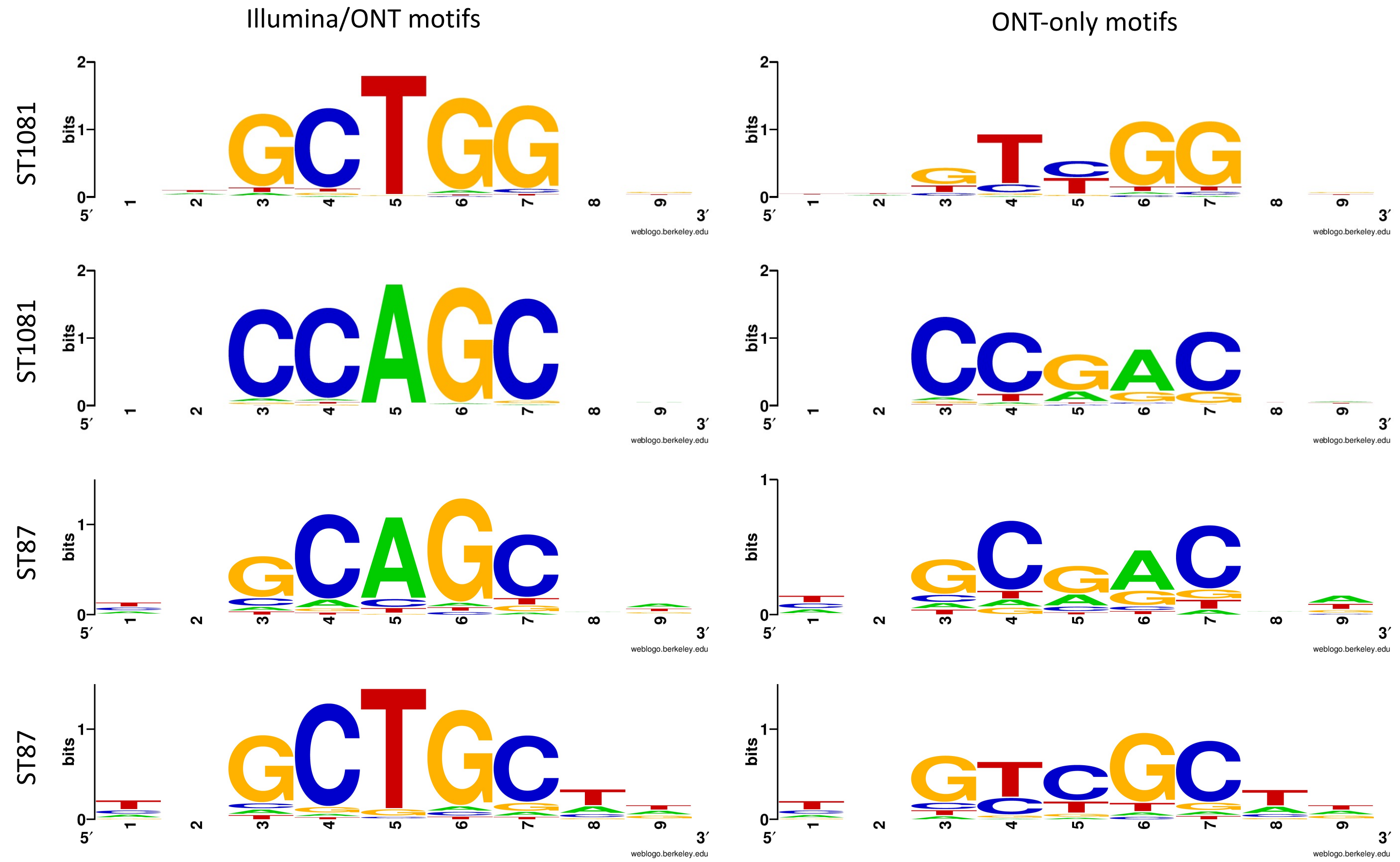

Supplementary Figure 6. Comparison of the modification motifs for the ST1081 and ST81 strains separately on the hybrid ONT/Illumina and ONT-only genomes. Modifications on the ST1081 are centered on the GCTCC/CCAGC motifs, while those of ST87 are on the GCAGC/GCTGC motifs.

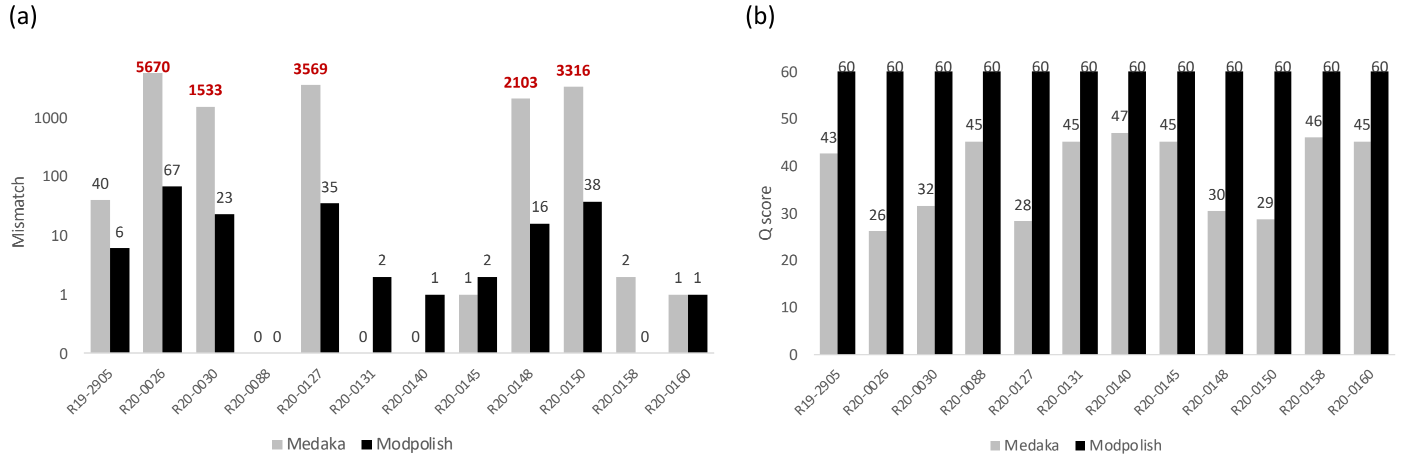

Supplementary Figure 7. Comparison of the mismatches and Q scores of Medaka and Modpolish in the 12 *Listeria* strains basecalled by Guppy 6.3.4 using the SUP model (grey: Medaka, black: Modpolish).

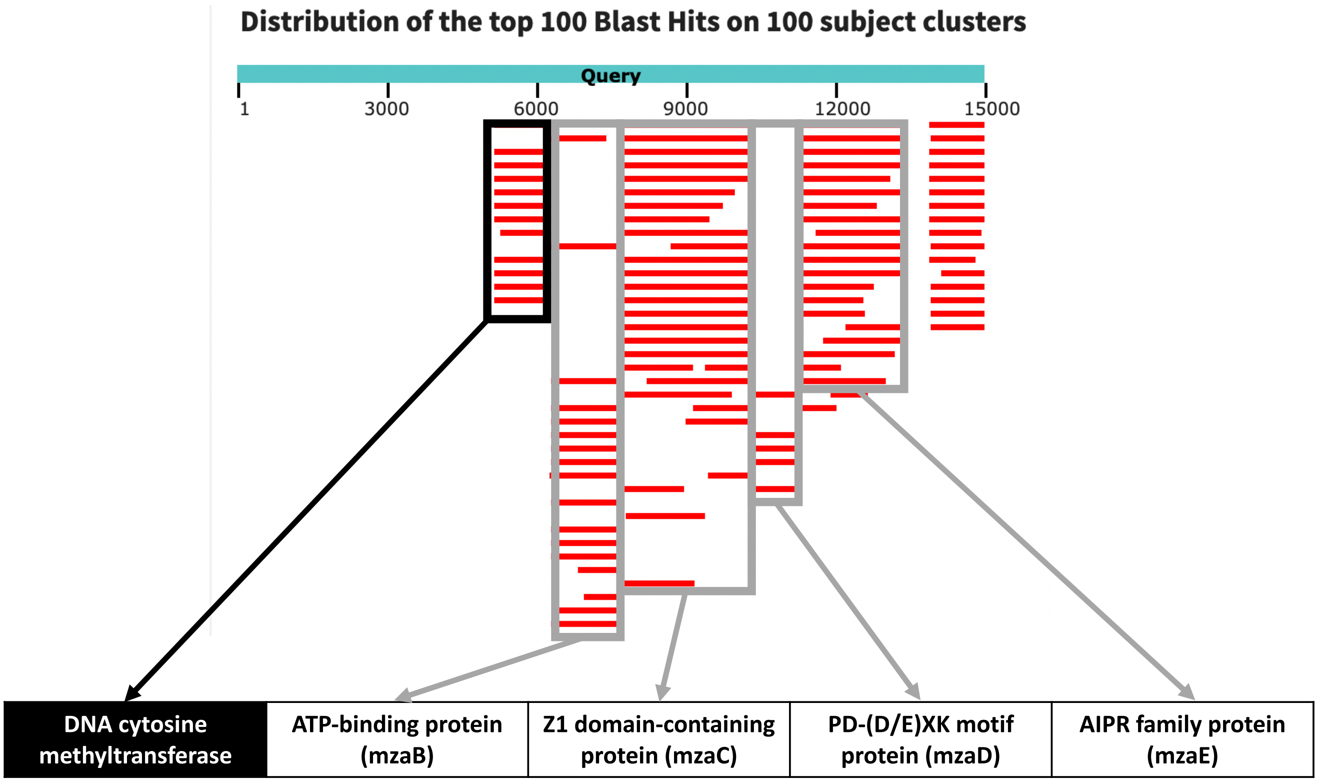

Supplementary Figure 8. BLAST search of the mza system members revealed a DNA cytosine methyltransferase followed by mzaB, mzaC, mzaD, and mzaE.

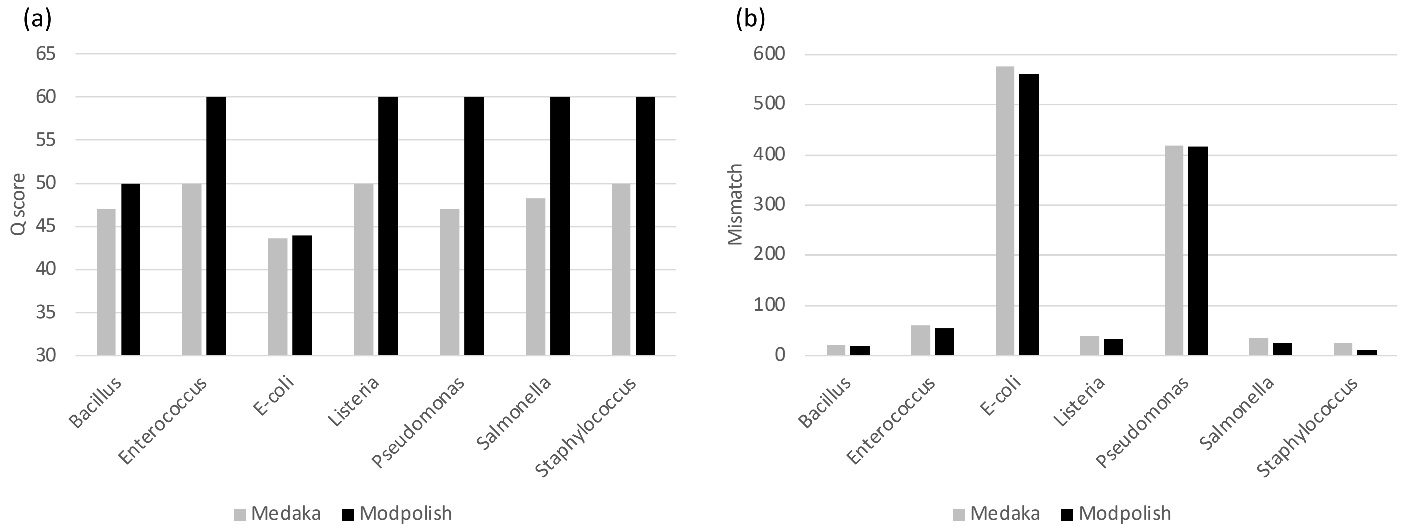

Supplementary Figure 9. Comparison of mismatches and Q scores of Medaka and Modpolish using the R10.4 simplex dataset (kit 14, Dorado v0.1.1, SUP) (grey: Medaka, black: Modpolish).

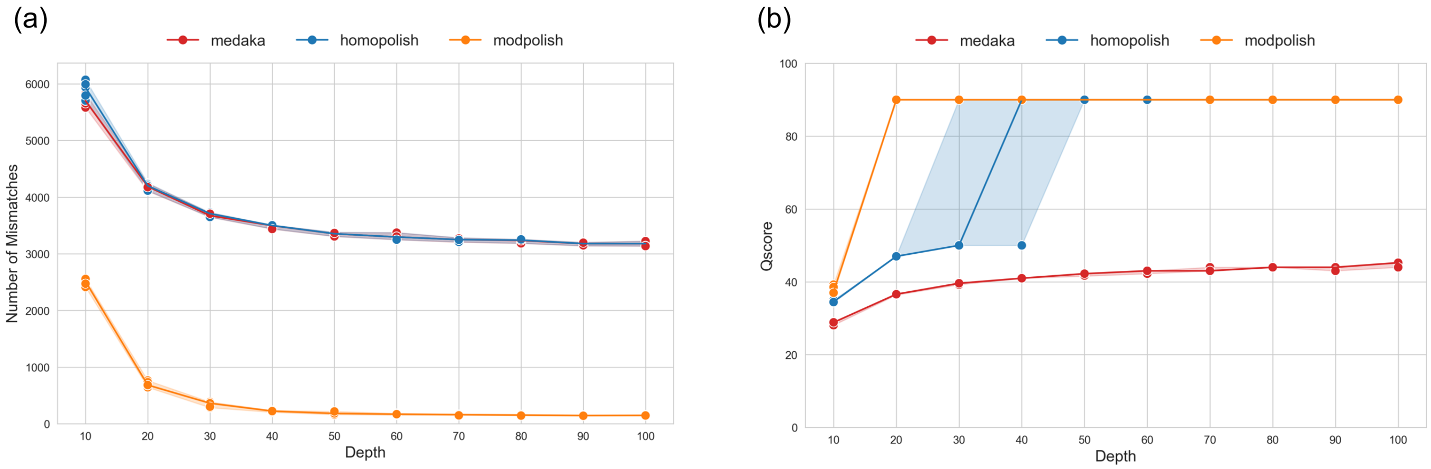

Supplementary Figure 10. The numbers of mismatches and Q scores in the *E coli* genomes polished by Medaka (red), Homopolish (blue), and Modpolish (orange). The shading spans the minimum and maximum values in five replicated experiments.

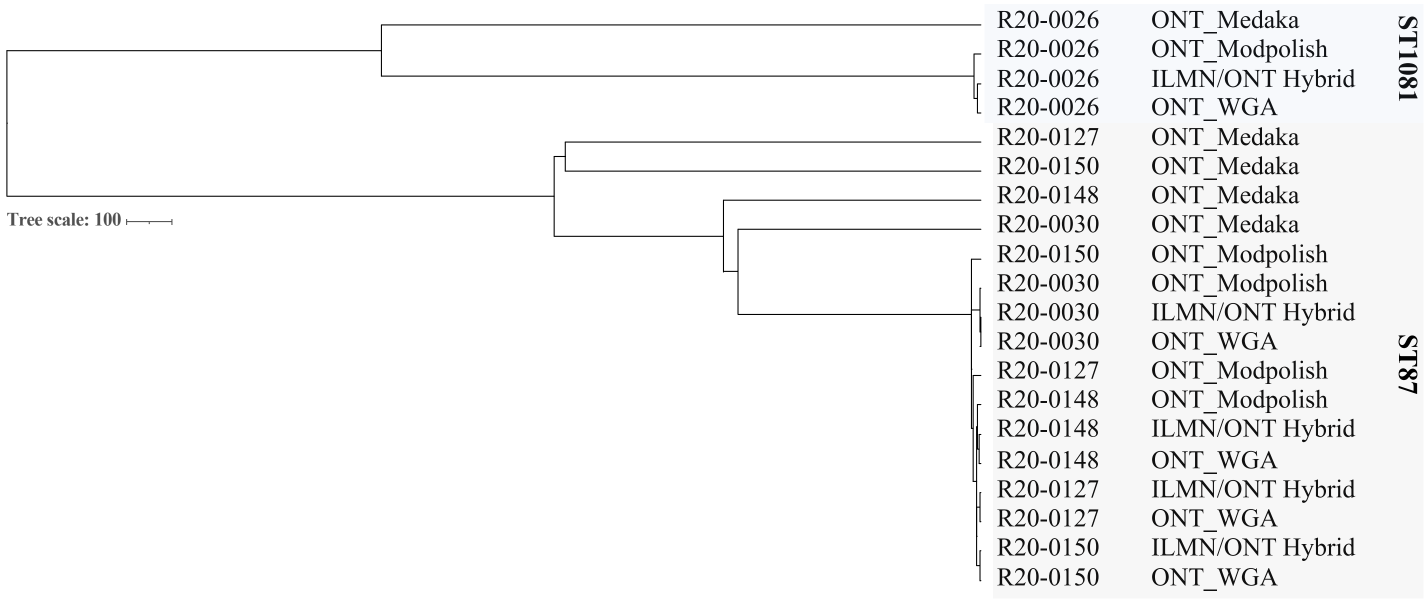

Supplementary Figure 11. Comparison of the cgMLST phylogeny of the five LQ *L monocytogene* strains among different methods. Each strain was ssembled by solely ONT (ONT_Medaka), WGA-demodified ONT (ONT_WGA), ONT with Modpolish (ONT_Modpolish), and hybrid ONT/Illumina genomes (ILMN/ONT Hybrid).

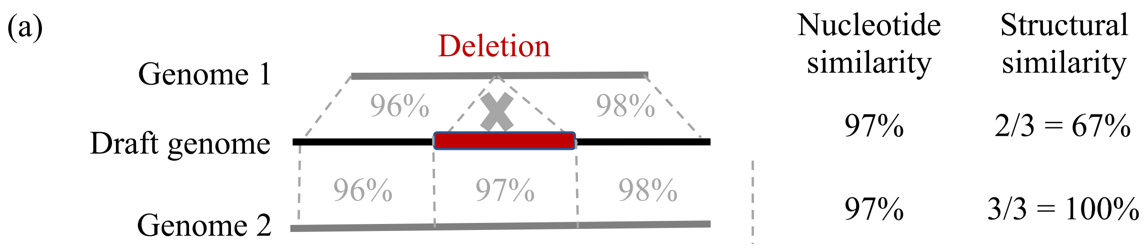

(b)

		1	2	3	4	5	6	7	8	9	10
True genome		A	G	C	T	G	**C**	**G**	T	G	G
Draft genome		A	G	C	T	G	**T**	**T**	T	G	G
Homologous alleles	A	20			10						1
	C			20		10	20				
	G		20			10		20		20	19
	T				10				20		
Read alleles	A	25			2	1	2	1	1		
	C			23		1	1	0			
	G		25			1	0	2		20	20
	T				18	17	17	17	19		
Average quality		20	20	20	16	12	8	12	16	20	20
Allele discordance		0	0	0	10	15	**15**	**15**	5	0	0
Homolog conservation		100	100	100	50	50	**100**	**100**	100	100	95

Supplementary Figure 12. Similarity computation and allele count statistics. (a) Comparision of structural and nucleotide similarities; (b) Illustration of homologous and read alleles pileup.

Supplementary Table 1. Illumina sequencing statistics of the 12 samples.

	Read Number	Max Length	N50 Length	Total Bases
**R19-2905**	935808	301	301	251516220
**R20-0026**	681360	301	301	199375540
**R20-0030**	765356	301	301	223843041
**R20-0088**	792890	300	300	211480191
**R20-0127**	1143744	301	301	310918904
**R20-0131**	808834	300	300	217882330
**R20-0140**	1084336	301	301	296601904
**R20-0145**	1046572	301	301	283615978
**R20-0148**	1340102	300	300	359675819
**R20-0150**	1153082	301	301	310089709
**R20-0158**	1254996	301	301	343552792
**R20-0160**	1364554	301	301	370035262

Supplementary Table 2. ONT sequencing statistics of the 12 samples (R9.4, SUP).

	Read Number	Max Length	N50 Length	Total Bases
**R19-2905**	103489	113264	6988	311272973
**R20-0026**	109179	133103	8776	409731944
**R20-0030**	318211	109539	4237	658332239
**R20-0088**	95611	110293	9450	345134126
**R20-0127**	136705	113937	8906	552032094
**R20-0131**	100549	108202	8433	381762248
**R20-0140**	377099	74028	5124	990811868
**R20-0145**	291672	180955	5879	877310303
**R20-0148**	265358	80144	4282	589230943
**R20-0150**	90990	107273	9529	364792267
**R20-0158**	111136	127984	9827	438704763
**R20-0160**	124026	106338	10960	593804240

Supplementary Table 3. Assembly statistics of ONT-only sequencing (R9.4, SUP).

	Contig Number	Max Length	N50 Length	Total Bases
**R19-2905**	5	2408977	2408977	3139752
**R20-0026**	2	2941441	2941441	2947228
**R20-0030**	2	2953610	2953610	2958812
**R20-0088**	3	2949122	2949122	3074902
**R20-0127**	2	2992895	2992895	2998908
**R20-0131**	2	2952984	2952984	2958875
**R20-0140**	4	1802146	1802146	2932180
**R20-0145**	2	2895673	2895673	2901698
**R20-0148**	2	2912509	2912509	2917601
**R20-0150**	4	3015129	3015129	3070241
**R20-0158**	2	2945198	2945198	2951211
**R20-0160**	2	2986867	2986867	2992875

Supplementary Table 4. Assembly statistics of hybrid ONT/Illumina sequencing

	Contig Number	Max Length	N50 Length	Total Bases
**R19-2905**	1	3160104	3160104	3160104
**R20-0026**	1	2941412	2941412	2941412
**R20-0030**	1	2953630	2953630	2953630
**R20-0088**	2	2949137	2949137	3009037
**R20-0127**	1	2992871	2992871	2992871
**R20-0131**	1	2953003	2953003	2953003
**R20-0140**	1	2953885	2953885	2953885
**R20-0145**	1	2895698	2895698	2895698
**R20-0148**	1	2912653	2912653	2912653
**R20-0150**	1	3015149	3015149	3015149
**R20-0158**	1	2945208	2945208	2945208
**R20-0160**	1	2986889	2986889	2986889

Supplementary Table 5. Quality assessment of ONT-only genomes polished by Medaka and Homopolish (R9.4, SUP).

	**Mismatch**	**Insertion**	**Deletion**	**Q score**
**R19-2905**	40	20	23	47
**R20-0026**	5670	304	166	27
**R20-0030**	1533	10	17	34
**R20-0088**	0	15	16	50
**R20-0127**	3569	64	20	29
**R20-0131**	0	12	24	50
**R20-0140**	0	14	16	60
**R20-0145**	1	7	18	60
**R20-0148**	2103	15	7	32
**R20-0150**	3316	62	62	29
**R20-0158**	2	11	28	50
**R20-0160**	1	7	10	60

Supplementary Table 6. Numbers of 5mC and 6mA modifications identified by Megalodon.

	**5mC**	**6mA**
**R19-2905**	343754	189433
**R20-0026**	295057	142989
**R20-0030**	339836	218745
**R20-0088**	218745	155818
**R20-0127**	268977	114577
**R20-0131**	263553	141580
**R20-0140**	324727	223724
**R20-0145**	336799	196019
**R20-0148**	245296	98068
**R20-0150**	304172	147307
**R20-0158**	262102	139438
**R20-0160**	260236	126020

Supplementary Table 7. ONT sequencing statistics of the 12 WGA-demodified samples.

	Read Number	Max Length	N50 Length	Total Bases
**R19-2905**	188066	68252	4008	381295211
**R20-0026**	161346	74725	3998	323072380
**R20-0030**	225282	80954	3778	429041543
**R20-0088**	295820	98758	3760	551208279
**R20-0127**	351965	105728	3754	660699365
**R20-0131**	361034	94318	4085	744673304
**R20-0140**	320358	90561	3452	564265875
**R20-0145**	227362	64678	3783	431307951
**R20-0148**	342611	75934	3653	633592810
**R20-0150**	224242	57070	3896	443942733
**R20-0158**	218499	73549	3798	398753574
**R20-0160**	183849	90537	3810	338761482

Supplementary Table 8. Assembly statistics of WGA-demodified ONT sequencing

	Contig Number	Max Length	N50 Length	Total Bases
**R19-2905**	6	2408926	2408926	3163109
**R20-0026**	3	1498066	1498066	2947083
**R20-0030**	6	1375174	1368605	2970480
**R20-0088**	3	2460960	2460960	2966134
**R20-0127**	4	2303825	2303825	3011536
**R20-0131**	2	1519287	1519287	2946950
**R20-0140**	8	1104249	942128	2968716
**R20-0145**	5	1214009	917883	2907726
**R20-0148**	5	1585007	1585007	2944803
**R20-0150**	3	1555559	1555559	2981832
**R20-0158**	1	2974086	2974086	2974086
**R20-0160**	7	1290018	1264214	3038421

Supplementary Table 9. Quality assessment of WGA-demodified ONT genomes

	**Mismatch**	**Insertion**	**Deletion**	**Q score**
**R19-2905**	12	281	23	60
**R20-0026**	16	6456	30	53
**R20-0030**	17	10	6033	60
**R20-0088**	47	35	2065	50
**R20-0127**	6	17	35	60
**R20-0131**	17	40	31	50
**R20-0140**	44	2630	27	53
**R20-0145**	20	10	17	60
**R20-0148**	23	7917	23	60
**R20-0150**	14	14	6023	60
**R20-0158**	16	19	16	50
**R20-0160**	49	30	42	60

Supplementary Table 10. Quality assessment of ONT genomes corrected by Modpolish.

	**Mismatch**	**Insertion**	**Deletion**	**Q score**
**R19-2905**	6	7	14	60
**R20-0026**	67	20	21	50
**R20-0030**	23	5	6	60
**R20-0088**	0	51	23	50
**R20-0127**	35	41	16	50
**R20-0131**	2	2	3	60
**R20-0140**	1	13	18	60
**R20-0145**	2	1	1	60
**R20-0148**	16	14	8	60
**R20-0150**	38	28	15	50
**R20-0158**	0	17	0	60
**R20-0160**	1	1	0	60

Supplementary Table 11. The coverage, true positive (TP), true negative (TN), false positive (FP), false negative (FN), sensitivity, and specificity of Modpolish corrections on the 12 Listeria strains.

	Coverage	TP	TN	FP	FN	Sensitivity	Specificity
R19-2905	121	43	3121746	0	1	97.73%	100.00%
R20-0026	110	5226	2936114	0	51	99.03%	100.00%
R20-0030	145	1122	2952319	5	10	99.12%	100.00%
R20-0088	183	8	3008962	0	0	100.00%	100.00%
R20-0127	221	3374	2989449	0	30	99.12%	100.00%
R20-0131	252	1	2952987	3	0	100.00%	100.00%
R20-0140	191	1	2927143	1	0	100.00%	100.00%
R20-0145	149	2	2895648	1	1	66.67%	100.00%
R20-0148	218	2698	2909920	0	15	99.45%	100.00%
R20-0150	147	3159	3011849	0	31	99.03%	100.00%
R20-0158	135	4	2945193	0	0	100.00%	100.00%
R20-0160	113	7	2986861	0	0	100.00%	100.00%

Supplementary Table 12. The true positive (TP), true negative (TN), false positive (FP), false negative (FN), sensitivity, and specificity of Modpolish on the Zymo R9.4 datasets.

Species	TP	TN	FP	FN	Sensitivity	Specificity
B subtilis	81	4044779	0	40	66.94%	100.00%
E faecalis	87	2837148	3	62	58.39%	100.00%
E coli	28	4746533	17	556	4.79%	100.00%
L monocytogenes	80	2991410	0	37	68.38%	100.00%
P aeruginosa	52	6790391	0	435	10.68%	100.00%
S enterica	28	4738374	2	25	52.83%	100.00%
S aureus	294	2718314	1	94	75.77%	100.00%

Supplementary Table 13. The true positive (TP), true negative (TN), false positive (FP), false negative (FN), sensitivity, and specificity of Modpolish on the Zymo R10.4 datasets.

Species	TP	TN	FP	FN	Sensitivity	Specificity
B subtilis	139	4044364	0	67	67.48%	100.00%
E faecalis	119	2836313	0	127	48.37%	100.00%
E coli	0	4434442	0	1717	0.00%	100.00%
L monocytogenes	58	2978511	0	196	22.83%	100.00%
P aeruginosa	186	6785494	6	600	23.66%	100.00%
S enterica	12	4634787	67	1439	0.83%	100.00%
S aureus	220	2710621	0	88	71.43%	100.00%

Supplementary Table 14. Quality assessment of Zymo R9.4 dataset corrected by Modpolish.

	Mismatch	Insertion	Deletion	Q score
**Bacillus**	22	80	149	60
**Enterococcus**	59	46	0	60
**Escherichia**	556	59	21	48
**Lactobacillus**	348	26	1109	36.68
**Listeria**	44	532	832	50
**Salmonella**	24	18	9	60
**Staphylococcus**	13	8	77	60
**Pseudomonas**	428	3	118	60

Supplementary Table 15. Quality assessment of Zymo R10.4 dataset corrected by Modpolish.

Bacteria	Mismatch	Insertion	Deletion	Q score
**Bacillus**	19	80	149	60
**Enterococcus**	53	12	7	60
**Escherichia**	571	80	1485	46
**Listeria**	40	530	814	60
**Pseudomonas**	416	1	116	60
**Salmonella**	24	24	15	60
**Staphylococcus**	12	11	79	60

Supplementary Table 16. Comparison of contig numbers and N50 sizes of Illumina (ILMN), ONT WGS, and ONT WGA.

	Depth (X)			No. contigs			N50 Size		
	ILMN	ONT_WGS	ONT_WGA	ILMN	ONT_WGS	ONT_WGA	ILMN	ONT_WGS	ONT_WGA
R19-2905	69	96	114	123	5	6	374,210	2,408,977	2,408,926
R20-0026	56	136	104	59	2	3	537,650	2,941,441	1,498,066
R20-0030	66	217	137	97	2	6	562,341	2,953,610	1,368,605
R20-0088	60	112	177	57	3	3	340,520	2,949,122	2,460,960
R20-0127	89	180	209	72	2	4	483,391	2,992,895	2,303,825
R20-0131	62	126	237	38	2	2	492,260	2,952,984	1,519,287
R20-0140	86	327	180	43	4	8	477,305	1,802,146	942,128
R20-0145	83	296	140	26	2	5	1,452,144	2,895,673	917,883
R20-0148	105	197	205	25	2	5	1,461,890	2,912,509	1,585,007
R20-0150	88	118	139	38	4	3	562,314	3,015,129	1,555,559
R20-0158	100	145	127	31	2	1	503,930	2,945,198	2,974,086
R20-0160	106	194	106	31	2	7	543,215	2,986,867	1,264,214
