# Supplementary material for: Correcting modification-mediated errors in nanopore sequencing by nucleotide demodification and reference-based correction
Source: Commun Biol. 2023 Nov 29;6:1215. doi: 10.1038/s42003-023-05605-4 (PMC10687267; doi:10.1038/s42003-023-05605-4)
Supplement: Supplementary file 2 — Description of Additional Supplementary Files [file 42003_2023_5605_MOESM2_ESM.pdf]

Description of Additional Supplementary Files

File name: Supplementary Data 1
Description: The defending systems predicted by PADLOC and grouped by LQ or HQ strains. The mza and RM systems are highlighted in red and blue, respectively.

File name: Supplementary Data 2
Description: Source data used to plot Fig 1b-c, 1e, 2b-e, 3b-c, and 4.
