# Supplementary material for: Correcting modification-mediated errors in nanopore sequencing by nucleotide demodification and reference-based correction
Source: Commun Biol. 2023 Nov 29;6:1215. doi: 10.1038/s42003-023-05605-4 (PMC10687267; doi:10.1038/s42003-023-05605-4)
Supplement: Supplementary file 5 — Reporting Summary [file 42003_2023_5605_MOESM5_ESM.pdf]

nature portfolio

Corresponding author(s): Yao-Ting Huang

Last updated by author(s): Nov 3, 2023

# Reporting Summary

Nature Portfolio wishes to improve the reproducibility of the work that we publish. This form provides structure for consistency and transparency in reporting. For further information on Nature Portfolio policies, see our Editorial Policies and the Editorial Policy Checklist.

## Statistics

For all statistical analyses, confirm that the following items are present in the figure legend, table legend, main text, or Methods section.

| n/a | Confirmed | |
|---|---|---|
| ☐ | ☒ | The exact sample size (*n*) for each experimental group/condition, given as a discrete number and unit of measurement |
| ☐ | ☒ | A statement on whether measurements were taken from distinct samples or whether the same sample was measured repeatedly |
| ☐ | ☒ | The statistical test(s) used AND whether they are one- or two-sided<br>*Only common tests should be described solely by name; describe more complex techniques in the Methods section.* |
| ☐ | ☒ | A description of all covariates tested |
| ☐ | ☒ | A description of any assumptions or corrections, such as tests of normality and adjustment for multiple comparisons |
| ☐ | ☒ | A full description of the statistical parameters including central tendency (e.g. means) or other basic estimates (e.g. regression coefficient) AND variation (e.g. standard deviation) or associated estimates of uncertainty (e.g. confidence intervals) |
| ☐ | ☒ | For null hypothesis testing, the test statistic (e.g. $F$, $t$, $r$) with confidence intervals, effect sizes, degrees of freedom and $P$ value noted<br>*Give P values as exact values whenever suitable.* |
| ☐ | ☒ | For Bayesian analysis, information on the choice of priors and Markov chain Monte Carlo settings |
| ☐ | ☒ | For hierarchical and complex designs, identification of the appropriate level for tests and full reporting of outcomes |
| ☐ | ☒ | Estimates of effect sizes (e.g. Cohen's *d*, Pearson's *r*), indicating how they were calculated |

*Our web collection on statistics for biologists contains articles on many of the points above.*

## Software and code

Policy information about availability of computer code

| | |
|---|---|
| Data collection | The Nanopore reads for each isolate (in FAST5 file) were initially basecalled using Guppy v4.0.14 with the HAC model and later rebasecalled by v6.3.4 with the SUP model. In the ONT-only assembly, the sequences (in FASTQ file) were assembled using Flye (https://github.com/fenderglass/Flye)[26], then polished using the Racon (https://github.com/lbcb-sci/racon) [4], the Medaka (https://github.com/nanoporetech/medaka), and the Homopolish (https://github.com/ythuang0522/homopolish) [5]. |
| Data analysis | The Modpolish was implemented as a subcommand in the Homopolish package, which is freely available at (https://github.com/ythuang0522/homopolish/). |

For manuscripts utilizing custom algorithms or software that are central to the research but not yet described in published literature, software must be made available to editors and reviewers. We strongly encourage code deposition in a community repository (e.g. GitHub). See the Nature Portfolio guidelines for submitting code & software for further information.

## Data

Policy information about availability of data

All manuscripts must include a data availability statement. This statement should provide the following information, where applicable:

- Accession codes, unique identifiers, or web links for publicly available datasets
- A description of any restrictions on data availability
- For clinical datasets or third party data, please ensure that the statement adheres to our policy

The Illumina, ONT, and WGA ONT raw reads of the 12 isolates were deposited in the NCBI Short Read Archives (SRA) under the BioProject PRJNA839535 with SRA accession numbers: SRS1239568 and SRS13025957-SRS13025967.

## Research involving human participants, their data, or biological material

Policy information about studies with human participants or human data. See also policy information about sex, gender (identity/presentation), and sexual orientation and race, ethnicity and racism.

| | |
|---|---|
| Reporting on sex and gender | Not applicable. The bacterial isolates are collected from human samples in Taiwan with unknown sex and gender. |
| Reporting on race, ethnicity, or other socially relevant groupings | Not applicable. The bacterial isolates are collected from human samples in Taiwan with unknown race, ethnicity, or socially relevant groupings.. |
| Population characteristics | The bacterial isolates are collected from unkown human samples in Taiwan. |
| Recruitment | The bacterial isolates are collected from unknown human samples in Taiwan. |
| Ethics oversight | Not applicable. |

Note that full information on the approval of the study protocol must also be provided in the manuscript.

# Field-specific reporting

Please select the one below that is the best fit for your research. If you are not sure, read the appropriate sections before making your selection.

☒ Life sciences ☐ Behavioural & social sciences ☐ Ecological, evolutionary & environmental sciences

For a reference copy of the document with all sections, see nature.com/documents/nr-reporting-summary-flat.pdf

# Life sciences study design

All studies must disclose on these points even when the disclosure is negative.

| | |
|---|---|
| Sample size | We investigated the modification-mediated errors in 12 Listeria isolates from humans due to Nanopore sequencing. By comparing with Illumina sequencing, we identified modification-mediated errors are the leading cause of low-quality genomes produced by ONT. |
| Data exclusions | Not applicable |
| Replication | The 12 Listeria strains were repeated sequenced using two flowcells to justify the modification-mediated errors |
| Randomization | Not applicable |
| Blinding | Not applicable |

# Reporting for specific materials, systems and methods

We require information from authors about some types of materials, experimental systems and methods used in many studies. Here, indicate whether each material, system or method listed is relevant to your study. If you are not sure if a list item applies to your research, read the appropriate section before selecting a response.

## Materials & experimental systems

| n/a | Involved in the study |
| --- | --- |
| ☒ | ☐ Antibodies |
| ☒ | ☐ Eukaryotic cell lines |
| ☒ | ☐ Palaeontology and archaeology |
| ☒ | ☐ Animals and other organisms |
| ☒ | ☐ Clinical data |
| ☒ | ☐ Dual use research of concern |
| ☒ | ☐ Plants |

## Methods

| n/a | Involved in the study |
| --- | --- |
| ☒ | ☐ ChIP-seq |
| ☒ | ☐ Flow cytometry |
| ☒ | ☐ MRI-based neuroimaging |

# Plants

| | |
| --- | --- |
| Seed stocks | Not applicable |
| Novel plant genotypes | Not applicable |
| Authentication | Not applicable |
